# Direct Comparative Analyses of 10X Genomics Chromium and Smart-seq2

**DOI:** 10.1016/j.gpb.2020.02.005

**Published:** 2021-03-02

**Authors:** Xiliang Wang, Yao He, Qiming Zhang, Xianwen Ren, Zemin Zhang

**Affiliations:** 1 BIOPIC, Beijing Advanced Innovation Center for Genomics, and School of Life Sciences, Peking University, Beijing 100871, China; 2 Peking-Tsinghua Center for Life Sciences, Academy for Advanced Interdisciplinary Studies, Peking University, Beijing 100871, China

**Keywords:** Single-cell RNA sequencing, 10X, Smart-seq2, Bulk RNA-seq, Comparison

## Abstract

**Single-cell RNA sequencing** (scRNA-seq) is generally used for profiling transcriptome of individual cells. The droplet-based **10X** Genomics Chromium (10X) approach and the plate-based **Smart-seq2** full-length method are two frequently used scRNA-seq platforms, yet there are only a few thorough and systematic comparisons of their advantages and limitations. Here, by directly comparing the scRNA-seq data generated by these two platforms from the same samples of CD45^−^ cells, we systematically evaluated their features using a wide spectrum of analyses. Smart-seq2 detected more genes in a cell, especially low abundance transcripts as well as alternatively spliced transcripts, but captured higher proportion of mitochondrial genes. The composite of Smart-seq2 data also resembled **bulk RNA-seq** data more. For 10X-based data, we observed higher noise for mRNAs with low expression levels. Approximately 10%−30% of all detected transcripts by both platforms were from non-coding genes, with long non-coding RNAs (lncRNAs) accounting for a higher proportion in 10X. 10X-based data displayed more severe dropout problem, especially for genes with lower expression levels. However, 10X-data can detect rare cell types given its ability to cover a large number of cells. In addition, each platform detected distinct groups of differentially expressed genes between cell clusters, indicating the different characteristics of these technologies. Our study promotes better understanding of these two platforms and offers the basis for an informed choice of these widely used technologies.

## Introduction

After firstly introduced in 2009 [1], single-cell RNA sequencing (scRNA-seq) has dramatically influenced research fields ranging from cancer biology, stem cell biology to immunology [2], [3], [4], [5]. Compared with RNA-seq of bulk tissues with millions of cells, scRNA-seq provides an opportunity to analyze the composition of tissues/organs and the diversity of cellular states, as well as to detect rare cell types [6]. With the improvement of sequencing technologies, scRNA-seq is becoming robust and accessible for transcriptome analysis.

**Figure 1:**
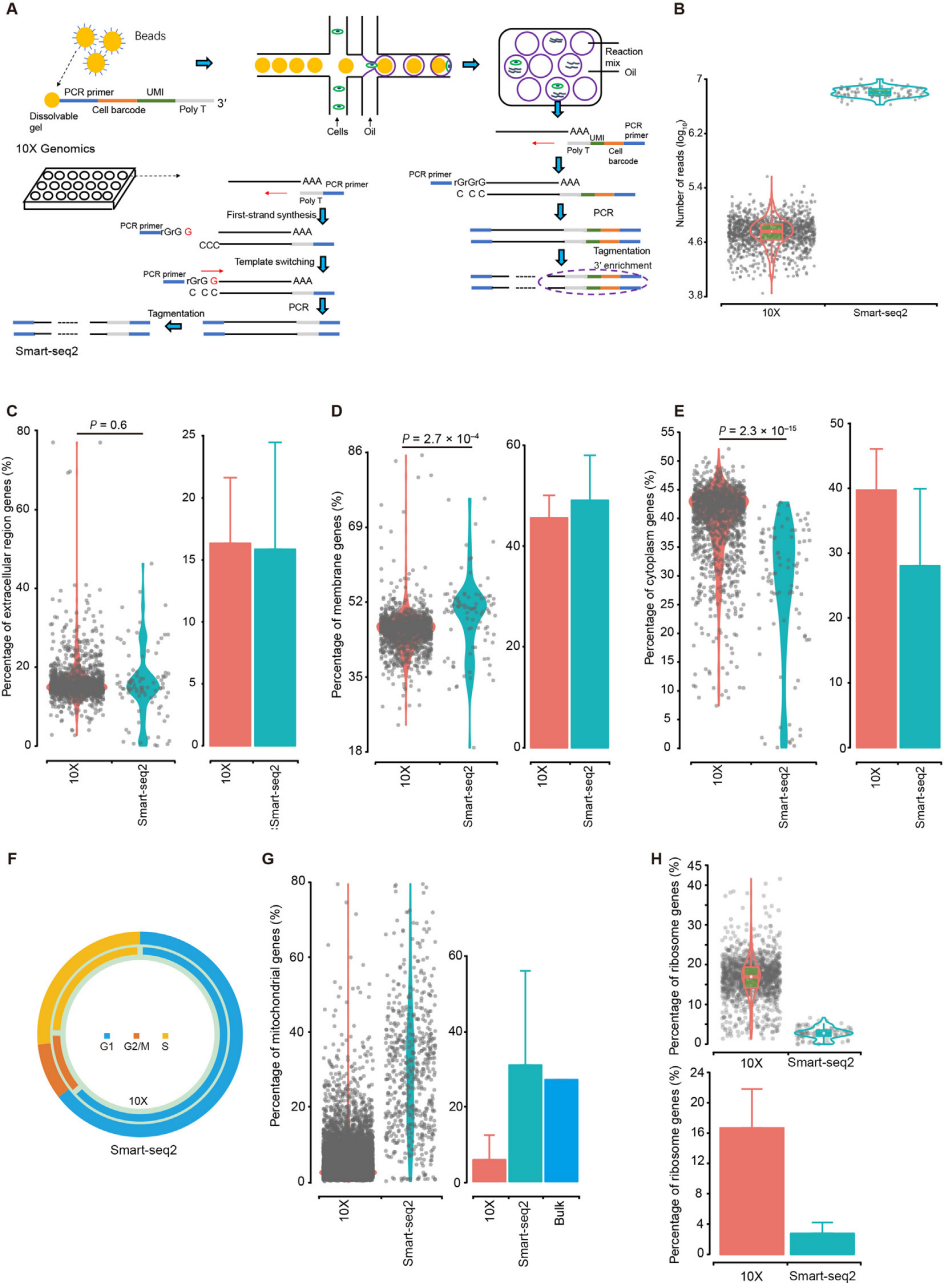
**Cell evaluation** **A.** The schematic diagrams of two scRNA-seq platforms. **B.** The total read number of each cell from LT.. **C.**–**E.** The proportion of reads of genes in the GO:0005576 “extracellular region” term (C), GO:0016020 “membrane” term (D), and GO:0005737 “cytoplasm” term (E) for cells from LT. **F.** The distribution of LT cells in the G1, G2/M, and S phases. **G.** and **H.** The proportion of reads of mitochondrial genes (G) and genes in the GO:0005840 “ribosome” term (H) for cells from LT. UMI, unique molecular identifier; LT, liver tumor.

Smart-seq2 [7] and 10X Genomics Chromium (10X; 10X Genomics, Pleasanton, CA) are two frequently-used scRNA-seq platforms (Figure 1A) [8], [9]. Smart-seq2 is based on microtiter plates [10], [11], where mRNA is separated and reverse transcribed to cDNA for each cell [12]. Reads mapped to a gene are used to quantify its abundance in every cell, and transcripts per kilobase million (TPM) is a common metric of expression normalization [13], [14]. By contrast, 10X is a droplet-based scRNA-seq technology, allowing genome-wide expression profiling for thousands of cells at once. The number of unique molecular identifiers (UMIs) is considered as a direct presentation of gene expression level [15]. Both TPM (Smart-seq2) and normalized UMI (10X) are analyzed to detect highly variable genes (HVGs), which are often used for either cellular phenotype classification or new subpopulation identification [16].

Smart-seq2 is one of the most successful methods for detecting single-cell gene expression with high robustness and reliability, and it is readily available to a wide community of researchers using few or no special instruments [17]. On the other hand, the most commonly used platform at present is the 10X platform. Although each platform has its own expected advantages and drawbacks based on the design of each method, there are only a few systematic comparisons of Smart-seq2 and 10X [17], [18]. Here, we applied these two technologies to the same samples, and directly compared the sensitivity (the probability to detect transcripts present in a single cell), precision (variation of the quantification), and power (subpopulation identification) of these two platforms.

## Results

### Data generation and evaluation

Our data were derived from two cancer patients. For the first patient, diagnosed to have hepatocellular carcinoma (HCC), we collected the liver tumor (LT) and its adjacent non-tumor (NT) tissues. For the second patient, diagnosed to have rectal cancer with liver metastasis, we collected both the primary tumor (PT) and the metastasized tumor (MT) tissues. For each sample, we used fluorescence activated cell sorting (FACS) to obtain CD45^−^ cells, and used both 10X and Smart-seq2 to perform scRNA-seq analysis. Following the standard experimental protocols, we obtained 10X data for 1338, 1305, 746, and 5282 cells for LT, MT, NT, and PT tissues, respectively, and obtained Smart-seq2 data for 94, 183, 189, and 135 cells for the corresponding tissues (Table S1). Bulk RNA-seq data of those four samples were also generated.

**Supplementary Figure S1:**
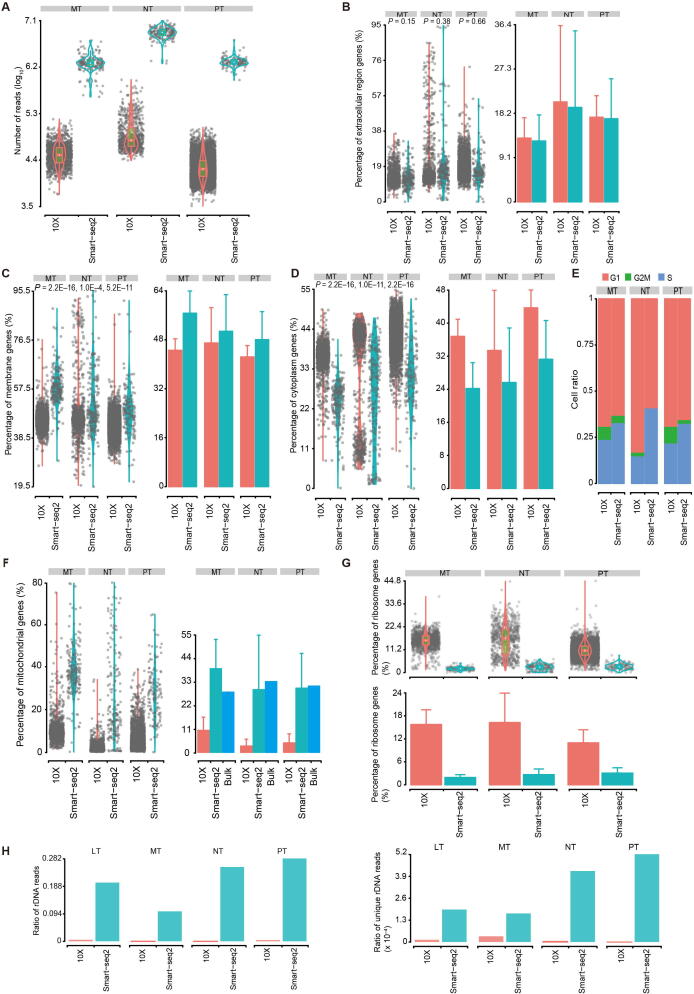
**Cell evaluation of other three samples A.** The total reads of each cell. The proportion of reads of genes in the GO:0005576 “extracellular region” term (**B**), GO:0016020 “membrane” term (**C**), and GO:0005737 “cytoplasm” term (**D**). **E.** The ratios of cells in the G1, G2M, and S phases. The proportion of reads of mitochondrial gene (**F**) and genes in GO:0005840 “ribosome” term (**G**). **H.** Read proportion of rDNA.

**Supplementary Figure S2:**
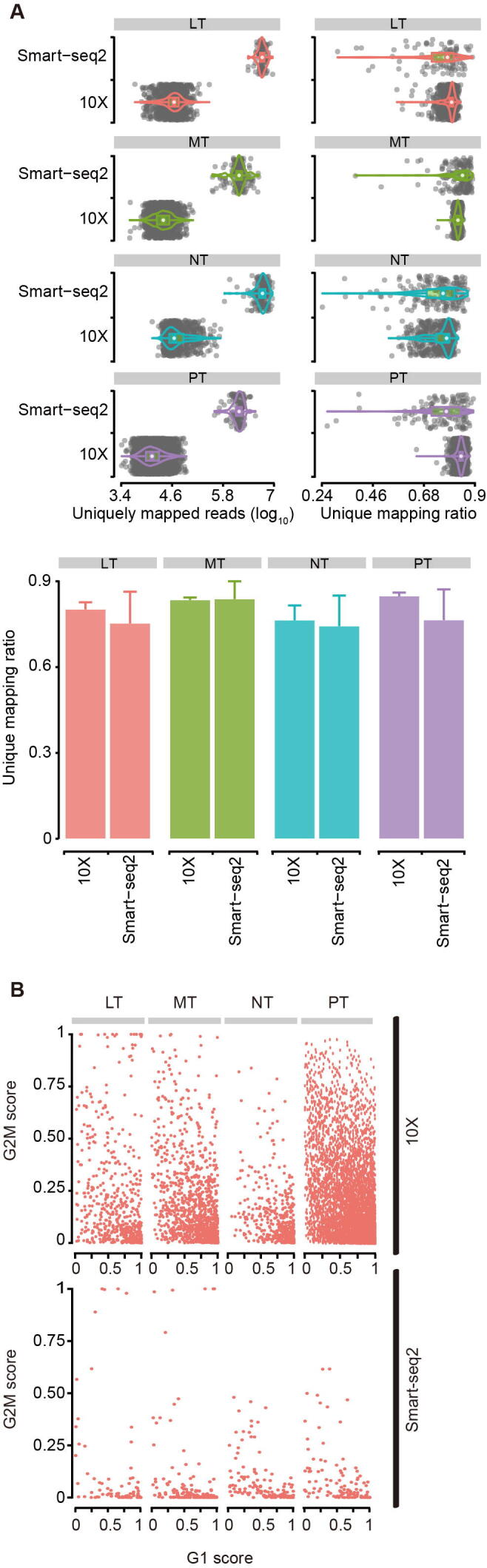
**Assessment of each cell A.** The number of unique mapped reads of each sample. B. Cell cycle phase scores of each cell.

We first examined the read counts for each cell derived from both platforms. The average total reads of each cell from Smart-seq2 were 6.2 M, 1.7 M, 6.3 M, and 1.7 M for LT, MT, NT, and PT, respectively, whereas 10X obtained relatively lower reads as follows: 59 K, 34 K, 92 K, and 20 K for the corresponding tissues (Figure 1B, Figure S1A). For transcriptome analysis, we followed conventional practice and selected uniquely mapped reads in the genome for downstream analysis. The number of uniquely mapped reads was nearly 9-fold higher in Smart-seq2 (Figure S2A). Although the 3′ ends have been reported to have higher homology than other parts of a gene, leading to increased level of multi-alignments [19], our results showed that the unique mapping ratios were similar, at approximately 80% for both datasets (Figure S2A).

As has been reported [20], damaged cells exhibited higher representation of genes in the “membrane” ontology category, but lower representation in the “extracellular region” and “cytoplasm” categories, when compared to high-quality cells. However, we did not observe obvious differences in the “extracellular region” category between those two scRNA-seq platforms (Figure 1C, Figure S1B). For Smart-seq2, the “membrane” category was over-represented (Figure 1D, Figure S1C) (all *P* < 10^−4^, two-sided *t*-test) and “cytoplasm” category under-represented (Figure 1E, Figure S1D) (all *P* < 10^−10^, two-sided *t*-test), implying more complete lysis of membranes.

Cell cycle has a major impact on gene expression [21], and is an important confounding factor of cell subpopulation classification [22]. We used an established method [23] to categorize cells into cell cycle phases (Figure S2B). The distributions of cells in G1, G2/M, and S phases were similar between the two platforms for all samples we studied (Figure 1F, Figure S1E).

### Higher proportion of mitochondrial genes for Smart-seq2 and ribosome-related genes for 10X

One metric we used to examine cell qualities is the ratio of reads mapped to the mitochondrial genome [24]. High levels of mitochondrial reads are indicative of poor quality, likely resulting from enhanced apoptosis and/or loss of cytoplasmic RNA from lysed cells [20]. Most reads from 10X contained a much lower abundance of mitochondrial genes ranging from 0%−15% of their total RNA. By contrast, the mitochondrial proportion from Smart-seq2 was 2.8–9.1 folds higher, at a level similar with bulk RNA-seq data (Figure 1G, Figure S1F). Such high proportions (an average of approximately 30%) were likely caused by more thorough disruption of organelle membranes by the Smart-seq2 and the standard bulk RNA-seq protocols than the relatively weak cell lysis procedure by 10X. Abnormally high proportion (such as > 50%) may reflect poor cell quality from Smart-seq2 in this study. However, caveats should be considered when examining mitochondrial genes, because naturally larger mitochondrial proportions can be expected from certain cells such as cardiomyocytes (58%−86%) [25] and those in apoptosis [20].

Ribosome-related genes (genes in the “ribosome” GO term) accounted for a large portion of detected transcripts by 10X, 2.6–7.2 folds higher than Smart-seq2 data (Figure 1H, Figure S1G). Indeed, 10X detected genes were enriched in the “ribosome” GO term, rather than ribosomal DNA (rDNA). The proportion of sequencing reads assigned to rDNA was only 0.03%−0.4% in 10X, significantly lower than that by Smart-seq2 (10.2%−28.0%). Few reads were uniquely mapped among those reads (Figure S1H); therefore, removing non-uniquely mapped reads was essential to minimize rDNA interference in Smart-seq2.

### 10X detected a higher proportion of lncRNA and Smart-seq2 identified more lncRNA as HVGs

**Figure 2:**
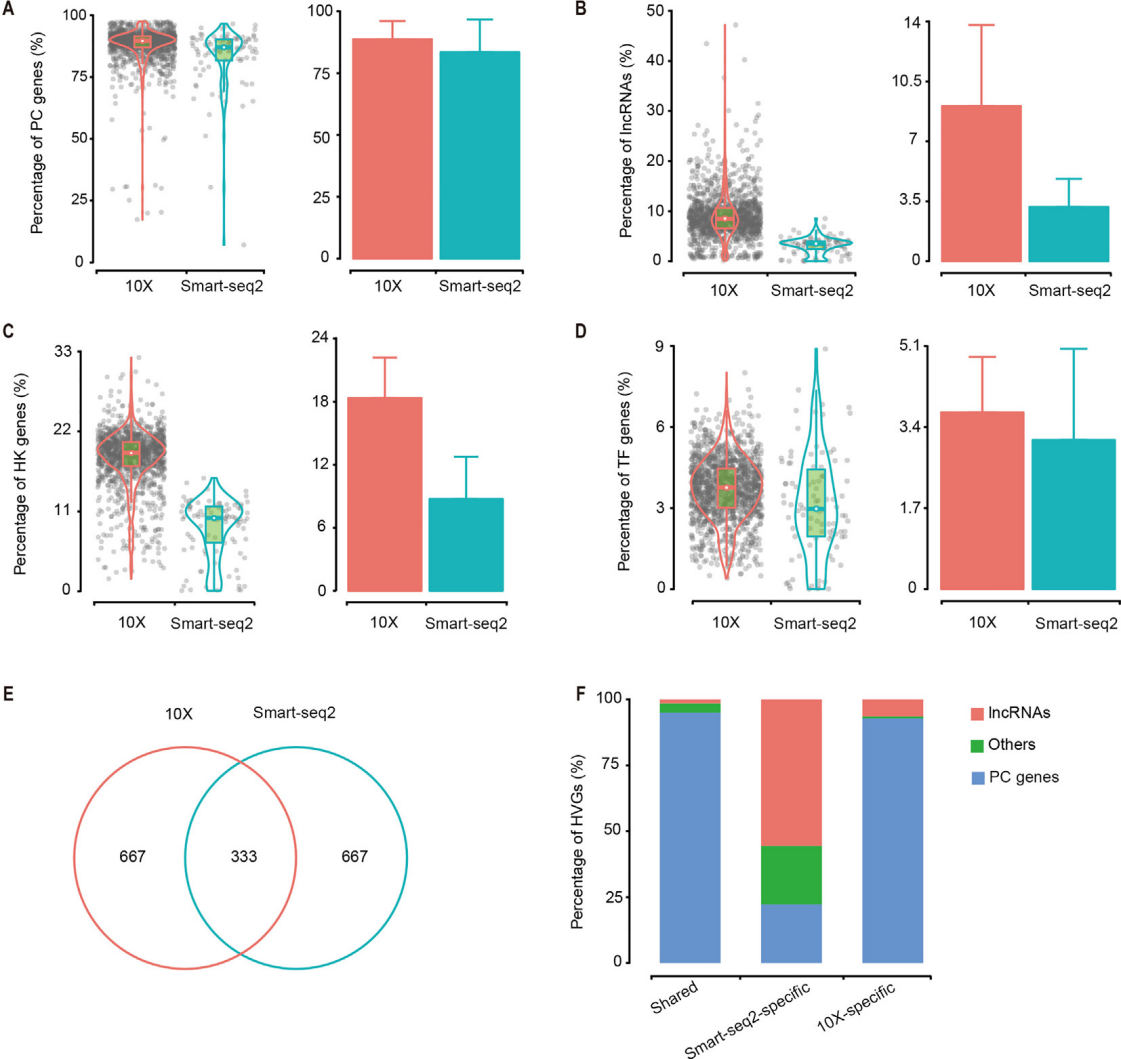
**Comparison of lncRNAs** **A.**–**D.** The ratio of reads of PC genes (A), lncRNAs (B), HK genes (C), and TF genes (D) detected in cells from LT. **E.** Overlap of top 1000 HVGs identified by 10X and Smart-seq2. **F.** Types of top 1000 HVGs. PC, protein-coding; lncRNA, long non-coding RNA; HK, house-keeping; TF, transcription factor; HVG, highly variable gene.

**Supplementary Figure S3:**
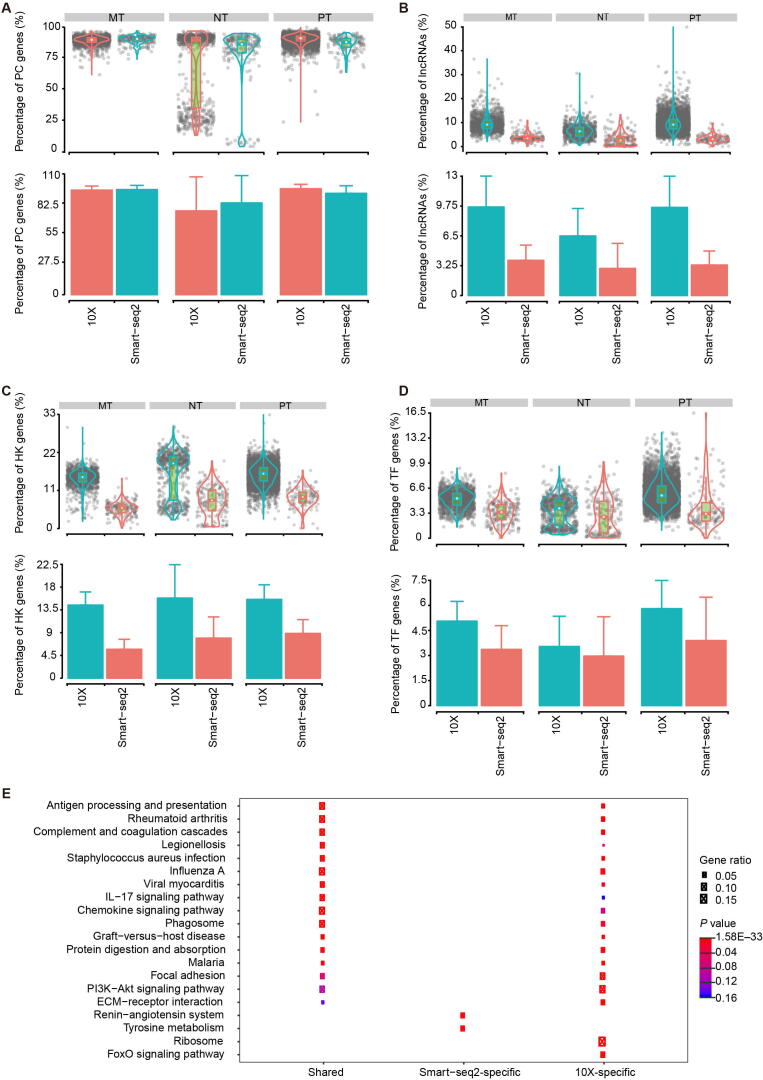
**Comparison of certain classes of genes** The expression proportion of PC genes (**A**), lncRNAs (**B**), HK genes (**C**), and TFs (**D**). **E.** KEGG enrichment results of 10X-specific, Smart-seq2-specific, and shared top 1000 HVGs. PC, protein-coding; HK, house-keeping; TF, transcription factor; HVG, highly variable gene.

Despite both Smart-seq2 and 10X followed the poly-A enrichment strategy, approximately 10%−30% of all detected transcripts were from non-coding genes (Figure 2A, Figure S3A), with long non-coding RNAs (lncRNAs) accounting for 2.9%−3.8% in Smart-seq2 and relatively higher (6.5%−9.6%) in 10X (Figure 2B, Figure S3B). In total, protein-coding (PC) genes and lncRNAs accounted for 80.5%−92.6% of all detected transcripts for Smart-seq2, and 77.4%−99.2% for 10X. Other classes of RNAs and/or their precursors were also detected with a great variance among experiments. Among PC genes, the proportions of house-keeping (HK) genes and transcriptional factor (TF) genes were 0.7–1.5 and 0.1–0.4 folds higher in 10X, respectively (Figure 2C and D, Figure S3C and D).

One common method to cluster in scRNA-seq datasets was to identify HVGs [26], [27], which assumes that large variation in gene expression among cells mainly comes from biological difference instead of technical batch effects. We selected top 1000 HVGs, and found 333 HVGs shared between two platforms (Figure 2E). Smart-seq2-specific HVGs only enriched in two KEGG pathways, while 10X-specific HVGs enriched in 34 pathways, including common pathways in cancer, such as “PI3K–Akt signaling pathway” (Figure S3E), suggesting that HVGs identified by 10X were more conducive to understanding biological difference among samples. PC genes accounted for 94.9%, 22.3%, and 92.8% of shared, Smart-seq2-specific, and 10X-specific HVGs, respectively (Figure 2F). Huge differences in HVGs come from the lncRNAs which have been previously shown to be expressed with biological function in scRNA-seq [19]. The enrichment of lncRNAs in Smart-seq2-specific HVGs, which resulted in a few enriched KEGG pathways, may be caused by specific sub-populations which predominantly expressed those lncRNAs [28], [29]. Less lncRNAs identified as HVGs in 10X may due to their much lower expression levels [30], [31], and higher dropout ratio.

### Smart-seq2 detected more genes and 10X identified more cell clusters

**Figure 3:**
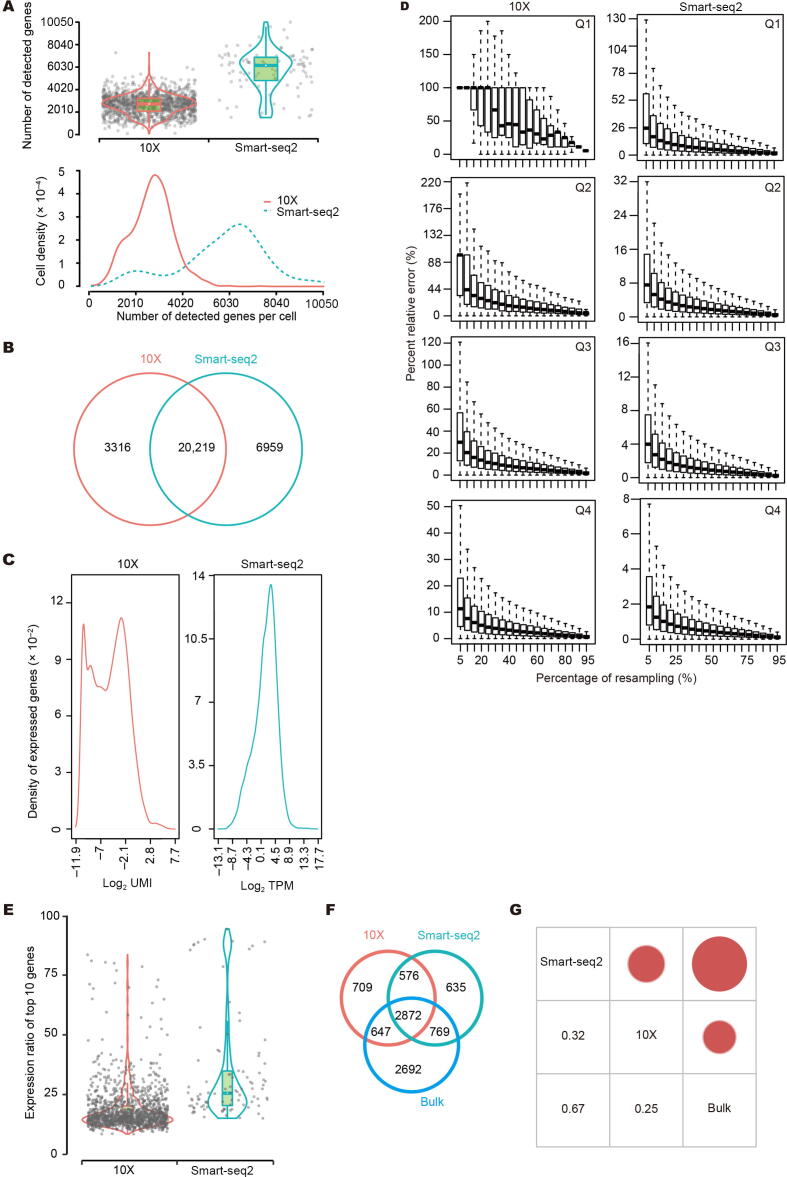
**Comparison of detected genes and their expression** **A.** The number of detected genes in every cell from LT. **B.** Overlap of all detected genes in cells from LT by 10X and Smart-seq2. **C.** Distribution of detected genes based on their expression levels in cells from LT. **D.** Saturation analysis by resampling a series of subsets of total reads from LT cells. **E.** Percentage of total counts assigned to the top 10 highly expressed genes in cells from LT. **F.** Overlap of the top 25% highly expressed genes in LT cells detected by 10X, Smart-seq2, and bulk RNA-seq. **G.** Correlation of expression of commonly detected genes among 10X, Smart-seq2, and bulk RNA-seq in cells from LT.

**Supplementary Figure S4:**
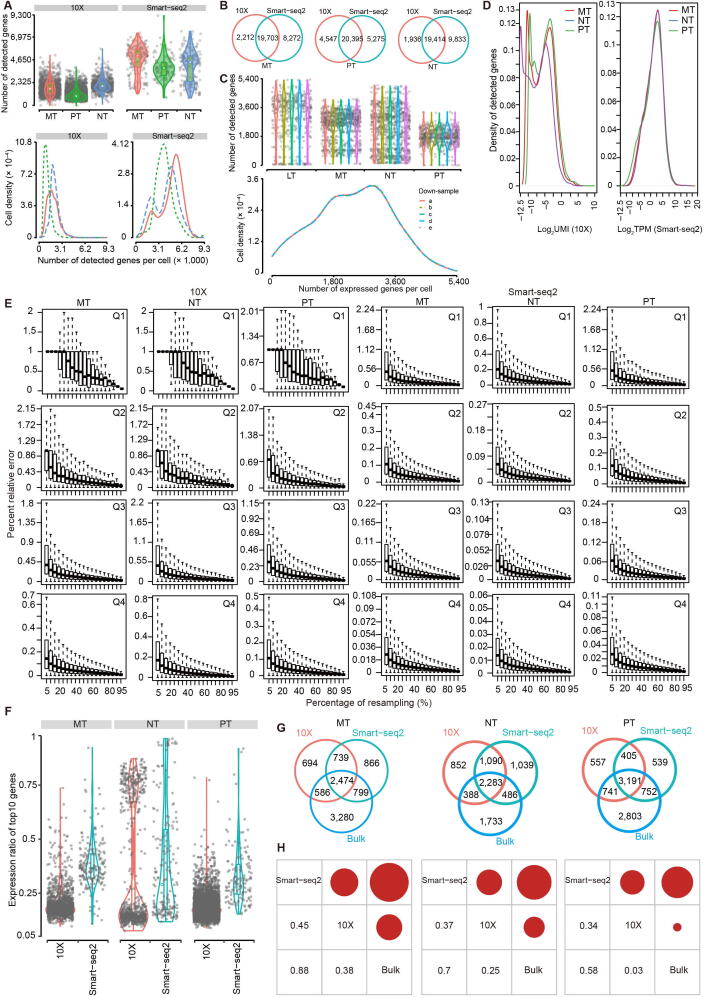
**Comparison of expression profiles A.** The number of detected genes in every cell. **B.** Overlap of all detected genes between 10X and Smart-seq2. **C.** The number of detected genes in every cell from Smart-seq2 with the artificially reduced reads number. **D.** Distribution of detected genes based on their expression levels. **E.** Saturation analysis. Y axis is “Percent Relative Error” which is used to measures how the RPKM estimated from subset of reads deviates from real expression level. **F.** Percentage of total counts assigned to the top 10 most highly abundant genes. **G.** Overlap of the top 25% highly expressed genes among 10X, Smart-seq2, and bulk RNA-seq. **H.** Correlation of commonly detected genes expression among 10X, Smart-seq2, and bulk RNA-seq.

We first assessed the sensitivity, represented as the number of discovered genes (TPM > 0 or UMI > 0) per cell [32]. Smart-seq2 had significantly higher sensitivity, capturing an average of 5713, 4761, 4079, and 3860 genes per cell for LT, MT, NT, and PT, respectively, compared to 2682, 1853, 2123, and 1104 genes for 10X, respectively (Figure 3A, Figure S4A). In total, more than 25,000 genes were covered from each sample by Smart-seq2; however, despite a magnitude more cells captured by 10X, approximately 20% genes were still dropped out (Figure 3B, Figure S4B). For a fair comparison, we down-sampled sequence reads from Smart-seq2 to a level that matched the sequencing depth in 10X. We still observed that higher number of genes detected per cell in the Smart-seq2 platform, with the artificially reduced read number (Figure S4C; Table S2), suggesting higher sensitivity of Smart-seq2. For detected genes, Smart-seq2 data showed a unimodal distribution with few lowly expressed genes detected in all cells. By contrast, 10X data showed an obvious bimodal distribution due to a large number of genes with near-zero expression (Figure 3C, Figure S4D), suggesting higher noise or random capture of mRNAs at very low expression level.

To examine the expression dynamic ranges covered by each platform, we determined the expression levels reaching saturation. All genes were divided into four quartiles by expression values. While sequencing depths of all four quartiles were saturated for Smart-seq2, only upper two quartiles were adequate for 10X (Figure 3D, Figure S4E), suggesting that Smart-seq2 has advantages in detecting genes at low expression levels. Meanwhile, the top 10 most highly expressed genes accounted for 33.0%−38.5% of total counts in Smart-seq2 and 18.4%−33.0% in 10X (Figure 3E, Figure S4F). Those 10 genes were dominated by mitochondrial genes, especially in Smart-seq2. Moreover, bulk RNA-seq data showed strikingly similar results to Smart-seq2 (Table S3).

We next determined if the two platforms covered different sets of genes. For any given sample, approximately 2/3 of genes present in the upper quartile were shared between the two platforms, leaving the remaining 1/3 genes distinct (Figure 3F, Figure S4G). Analysis of the distinct genes indicated that 5.6% of 10X-specific genes had full KEGG annotation, whereas only 2.7% of Smart-seq2-specific genes were annotated (Table S4). Thus, Smart-seq2 is better equipped at finding genes with unknown functions. In addition, Smart-seq2 shared more genes with bulk RNA-seq (Figure 3F, Figure S4G). Pearson correlation coefficient (PCC) between bulk RNA-seq and average Smart-seq2 single cell gene expression was higher (Figure 3G, Figure S4H), again showing more similarity between Smart-seq2 and bulk RNA-seq.

**Figure 4:**
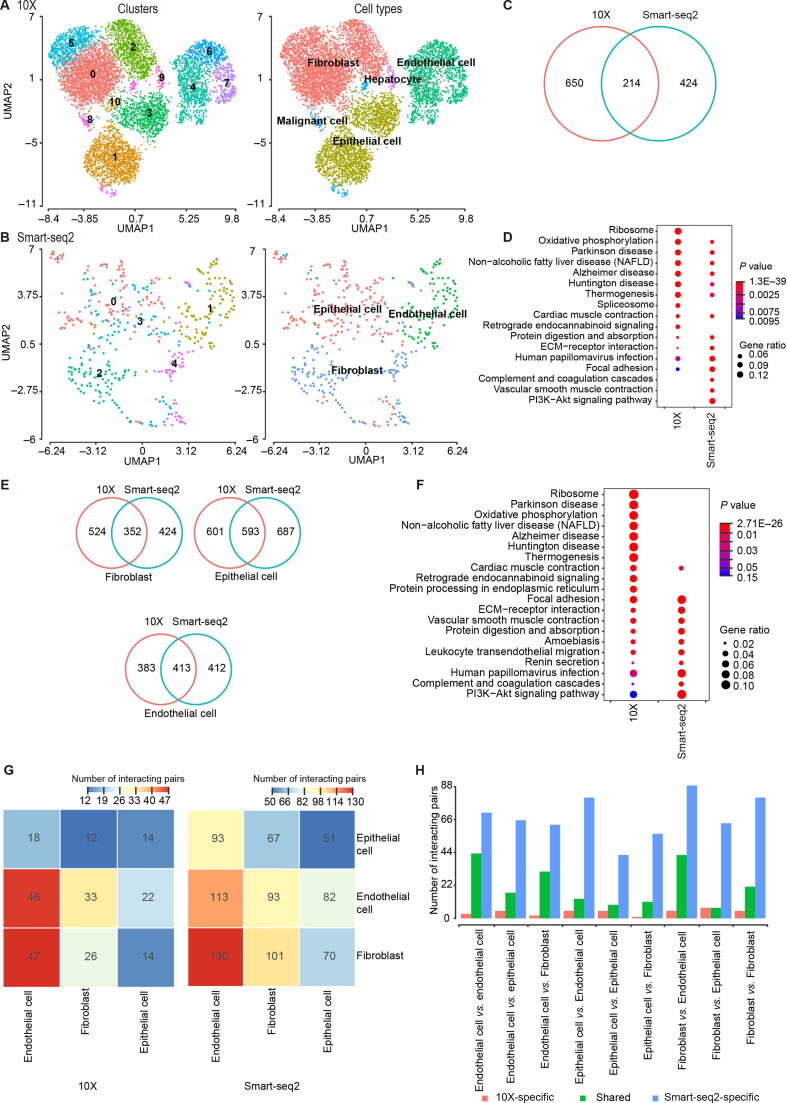
**Results of cellclustering and DEGs** **A.** and **B.** Cell clustering results for 10X (A) and Smart-seq2 (B). **C.** Overlap of DEGs of LT sample with other three samples identified by 10X and Smart-seq2. **D.** Comparison of KEGG enrichment results of LT sample. **E.** Overlap of DEGs of each cell type with remaining cell types between 10X and Smart-seq2. **F.** Comparison of KEGG enrichment results of fibroblasts. **G.** The total number of interaction pairs (*P* < 0.01) among cell types predicted from 10X and Smart-seq2, respectively. **H.** Comparison of interaction pairs (*P* < 0.01) among cell types predicted from 10X and Smart-seq2, respectively.

HVGs were used to cluster cells into putative subpopulations, which was one common objective for scRNA-seq research. Eleven clusters were identified in 10X using Seurat (version 2.3.4) [33] (Figure 4A). By applying conventional cell markers, those clusters were annotated as fibroblast, epithelial cell, endothelial cell, and two special cell types: “hepatocyte” and “malignant cell”, which highly expressed their respective markers, such as *ALB* and *SERPINA1* in hepatocyte, and *STMN1*, *H2AFZ*, *CKS1B*, and *TUBA1B* in malignant cells [34], [35] (Figure 4A). By contrast, only five clusters were identified in Smart-seq2 due to limited cell number, and these clusters were annotated as epithelial cell, endothelial cell, and fibroblast (Figure 4B). Four clusters of tumor fibroblasts were identified in 10X: cluster 0, cluster 2, cluster 5, and cluster 10 (Figure 4A). Cluster 0 cells showed fibroblast signatures (*RGS5* and *NDUFA4L2*), cluster 2 cells had strong expression of cancer associated fibroblast (CAF) markers (*LUM*, *SFRP4*, and *COL1A1*), and cluster 5 cells expressed myofibroblast markers (*MYH11*, *TAGLN*, and *ACTA2*). We also highlighted a fibroblast cluster (cluster 10) with a striking enrichment for mitochondrial genes (*MT-ND2*, *MT-CO3*, and *MT-CO2*). Smart-seq2 only identified two fibroblast subtypes, with cluster 2 cells expressing fibroblast signatures (*RGS5* and *NDUFA4L2*), and cluster 4 cells showing CAF markers (*LUM*, *DCN*, and *FBLN1*).

**Supplementary Figure S5:**
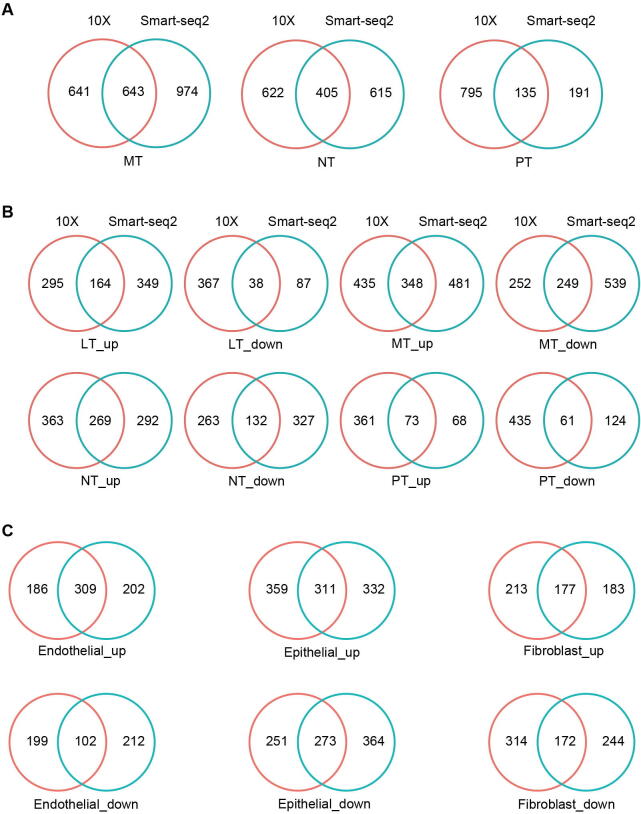
**Results of differentially expressed genes (DEGs) A.** Overlap of DEGs of remaining samples between Smart-seq2 and 10X. Overlap of Up-regulated and down-regulated DEGs for each sample (**B**) and each cell type (**C**) between Smart-seq2 and 10X.

**Supplementary Figure S6:**
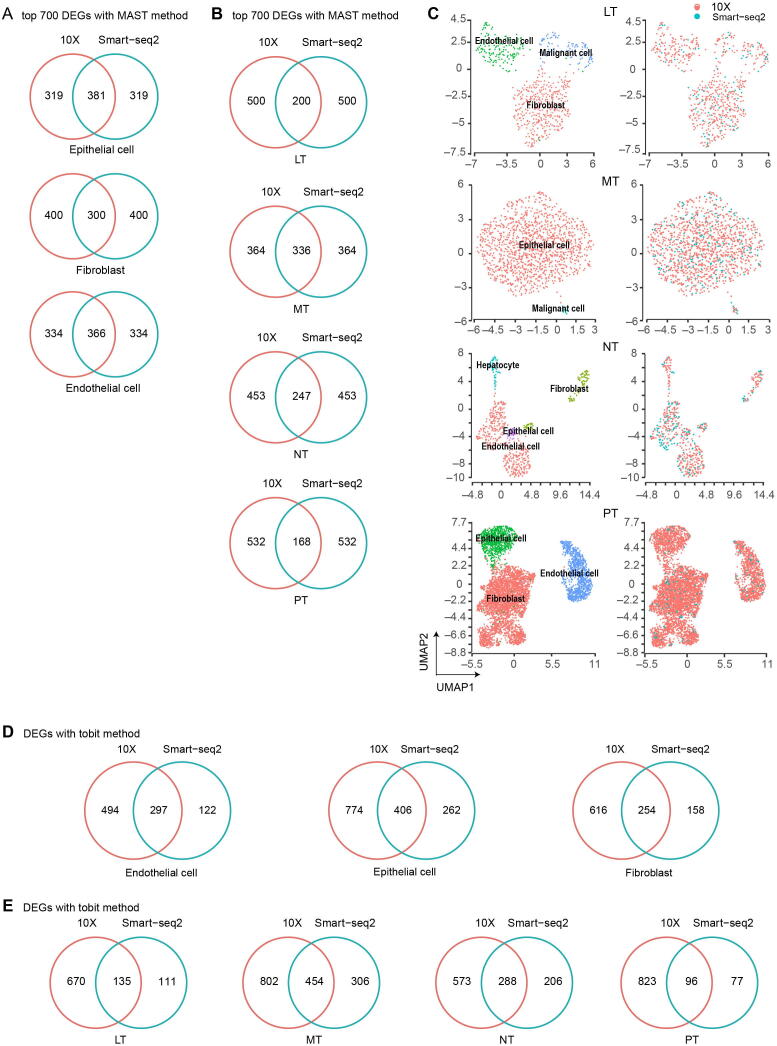
**Comparison of differentially expressed genes (DEGs)** Overlap of top700 DEGs with the smallest P value identified by 10X and Smart-seq2 among cell types (**A**) and samples (**B**). **C.** Cell clustering results with CCA used on an individual sample. Overlap of DEGs with the “tobit” method identified by 10X and Smart-seq2 among cell types (**D**) and samples (**E**).

We next examined if the two platforms covered different sets of differentially expressed genes (DEGs). We first identified DEGs within each sample compared to all other samples (Figure 4C, Figure S5A). 10X detected more DEGs in all samples expect for in MT, and less than 50% of total DEGs were shared between two platforms, leaving the remaining genes distinct. For example, 864 DEGs were identified between LT and other samples using 10X, and 20 KEGG pathways were enriched. Such numbers were 638 DEGs and 22 pathways for Smart-seq2, respectively. Only 214 DEGs (Figure 4C) and 11 pathways (Figure 4D) were shared. Considering up-regulated and down-regulated DEGs separately, less than 50% DEGs were shared between two platforms as well (Figure S5B). Moreover, we observed a few DEGs with conflicting directions (Table S5). We furthermore identified DEGs within one cell type compared to others (Figure 4E, Figure S5C). The same tendency was also found with several conflicted DEGs (Table S6). Exemplified with fibroblasts, 876 DEGs were identified between fibroblasts and other type cells, and enriched in 30 KEGG pathways using 10X, whereas 776 DEGs were identified and enriched in 23 pathways using Smart-seq2. Only 352 DEGs (Figure 4E) and 11 pathways (Figure 4F) were shared. To account for the different levels of gene detection by 10X and Smart-seq2, we also used the top 700 DEGs with the smallest *P* value for comparison and we obtained similar results (Figure S6A and B). We also performed canonical correlation analysis (CCA) on an individual sample analyzed by both platforms (Figure S6C). Following the DEGs (adjusted *P* < 0.01) identified from this analysis, we observed that 10X detected much more DEGs than Smart-seq2 (Table S7). For example, 963 DEGs were identified between fibroblasts and other type cells in LT in 10X, whereas 382 DEGs were identified in Smart-seq2. The aforementioned DEGs were detected using the “MAST” method, and we also used an alternative method “tobit” [36]. Their results were consistent to each other (Figure S6D and E), showing that differences of DEGs were mainly caused by platforms, instead of tools or selection cutoffs. In summary, the concordance between DEGs and enriched KEGG pathways by Smart-seq2 and 10X was limited, suggesting that the selection of platform indeed has an impact on the results. Notably, the “ribosome” pathway was spotted in 10X results (Figure 1H, Figure 4D and F, Figure S3E), showing gene detection bias of 10X.

To provide insights into the tumor-microenvironment characteristics derived from 10X and Smart-seq2, we compared the ability to predict potential cell–cell communication network from scRNA-seq datasets, which is an important but yet under-appreciated aspect of tumor microenvironment studies. We used those cell types (endothelial cell, fibroblast, and epithelial cell) that were detected by both platforms with CellPhoneDB (version 2.0) [37]. In spite of significant differences in the number of captured cells (Table S1), we observed that the total number of interactions (*P* < 0.01) among cell types predicted from Smart-seq2 data were at least 2 folds those from 10X-based prediction (Figure 4G and H). Thus, Smart-seq2 was a preferred platform to investigate cell–cell interaction. In addition, Smart-seq2-based prediction always found more unique interacting gene pairs, while almost all the 10X-predicted interacting pairs were covered by Smart-seq2. Our results demonstrated that richer expression information provided by Smart-seq2 data offered an advantage in cell–cell interaction analysis.

### 10X had a higher dropout ratio than Smart-seq2

**Figure 5:**
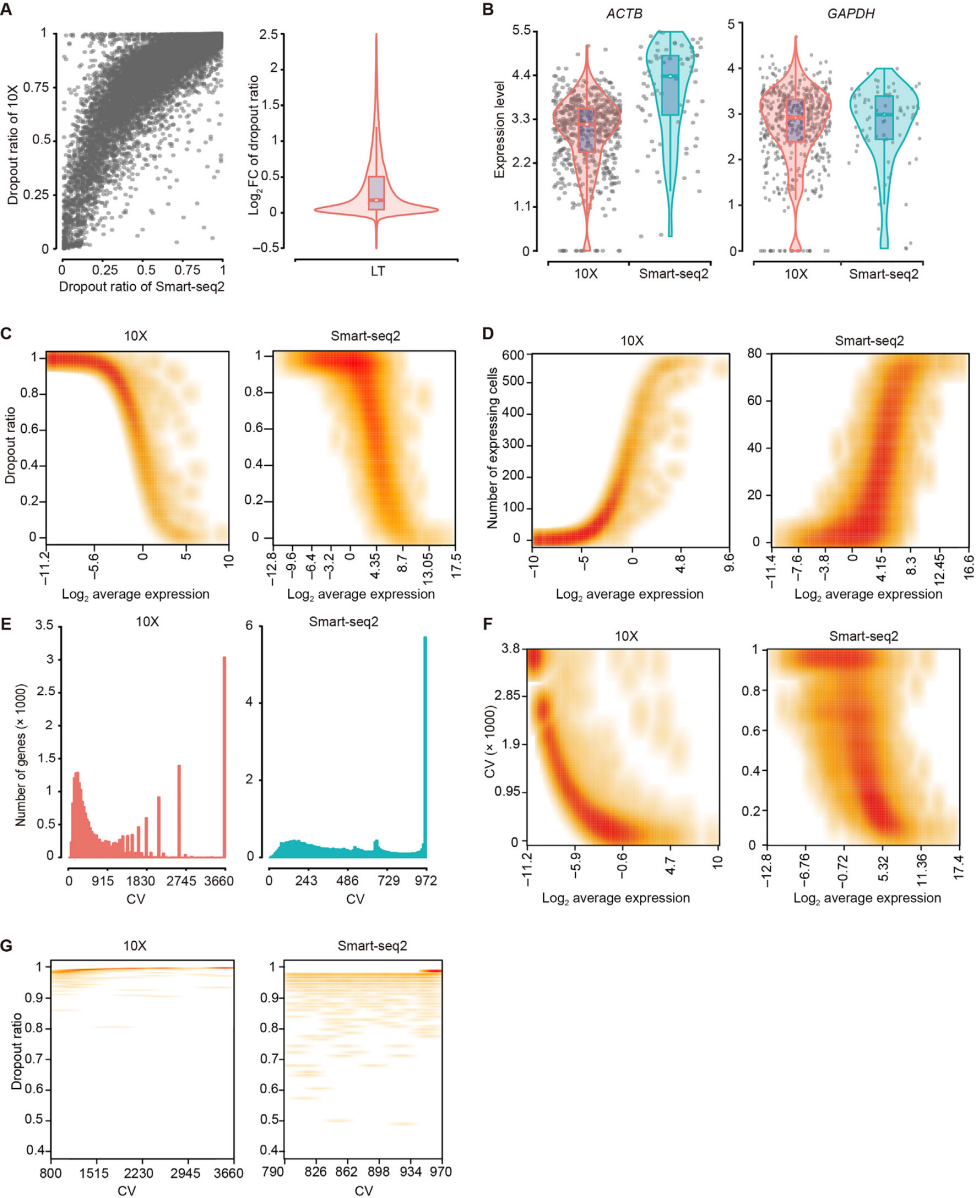
**Dropout assessment** **A.** Comparison of dropout ratios between 10X and Smart-seq2 in LT. **B.** Two examples of HK genes to show dropout events in LT. **C.** The relationship of dropout ratio and the average expression for each gene in LT. **D.** Number of expressing cells against the average expression of each gene in LT. **E.** CV distribution of each detected gene in LT. **F.** The relationship between CV and gene expression levels in LT. **G.** Dropout ratios of gene with CV more than 800 in LT. CV, coefficient of variation.

**Supplementary Figure S7:**
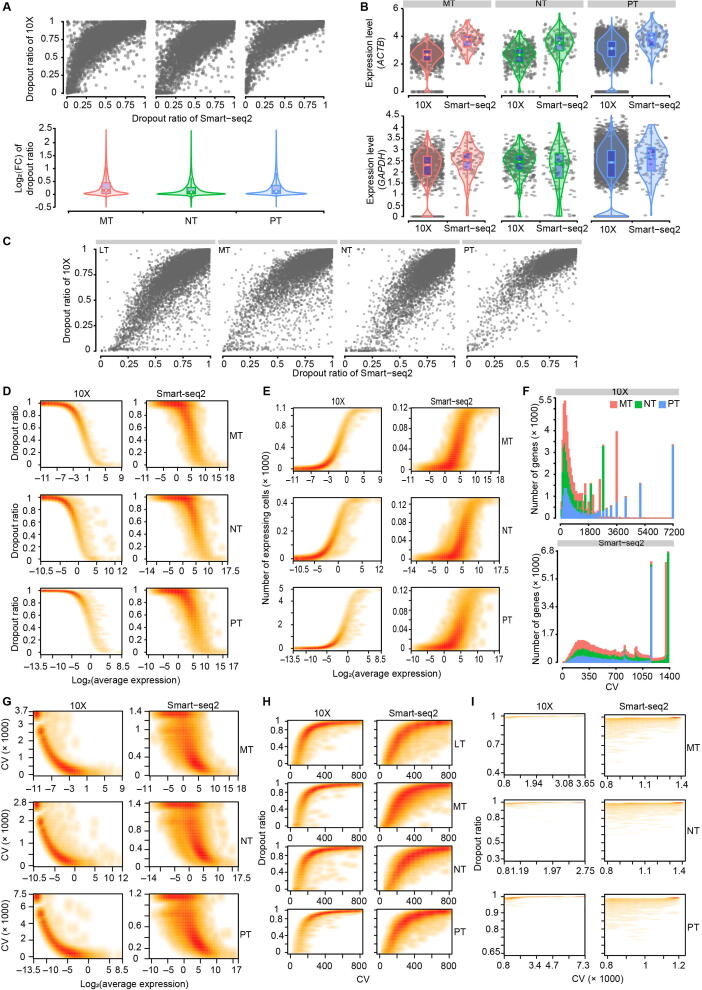
**Dropout events assessment of other three samples A.** Comparison of dropout ratios between 10X and Smart-seq2. **B.** Two examples of house-keeping genes. **C.** The relationships of dropout ratios and the average gene expression levels. **D.** Number of expressing cells against the average expression for each gene. **E.** CV distribution of each detected gene. **F.** The relationship between CV and gene expression levels. Dropout ratios of genes with CV less than 800 (**G**) and genes with CV more than 800 (**H**). CV, coefficient of variation.

Dropout events in scRNA-seq can result in many genes undetected and an excess of expression value of zero, leading to challenges in differential expression analysis [21], [38]. The average dropout ratios of majority genes in 10X were 1.3–1.4 folds those in Smart-seq2 for all samples tested (Figure 5A, Figure S7A). For example, the widely used HK gene *ACTB* had no dropout in Smart-seq2, whereas 2.8%−5.9% dropout ratios were observed in 10X (Figure 5B, Figure S7B). Similarly, *GAPDH* had dropout ratios of 0%−0.67% in Smart-seq2 but 4.2%−18.8% in 10X (Figure 5B, Figure S7B). However, after down-sampling of the single-cell data of Smart-seq2 to the similar read depth as achieved by 10X, we observed that 10X and Smart-seq2 had comparable dropout ratios (Figure S7C).

The frequency of dropout events was correlated to gene expression levels, which can be fitted by a modified non-linear Michaelis-Menten equation introduced in the M3Drop package (https://github.com/tallulandrews/M3Drop). Genes with lower expression levels had higher dropout ratios (Figure 5C, Figure S7D), consistent with a previous report [39]. Mitochondrial genes were the least likely to be dropped out, especially in Smart-seq2 (Table S8). In both platforms, genes with lower abundance were detected in smaller number of cells, and those genes could lead to higher noise, especially in 10X (Figure 5D, Figure S7E). Because genes with near-zero expression are noise without enough information for reliable statistical inference [40], removal of them may mitigate noise level and reduce the amount of computation without much loss of information.

We also found that the gene expression coefficient of variation (CV) across cells were associated with dropout ratios. 10X had more genes with large CV than Smart-seq2 (Figure 5E, Figure S7F). While genes with large CV generally had lower expression, especially for 10X (Figure 5F, Figure S7G), genes with larger CV also had higher dropout ratios (Figure S7H). For example, genes with CV larger than 800 had > 80% dropout ratios in Smart-seq2, near 100% of dropout in 10X (Figure 5G, Figure S7I).

### Difference in capture of gene structural information

**Figure 6:**
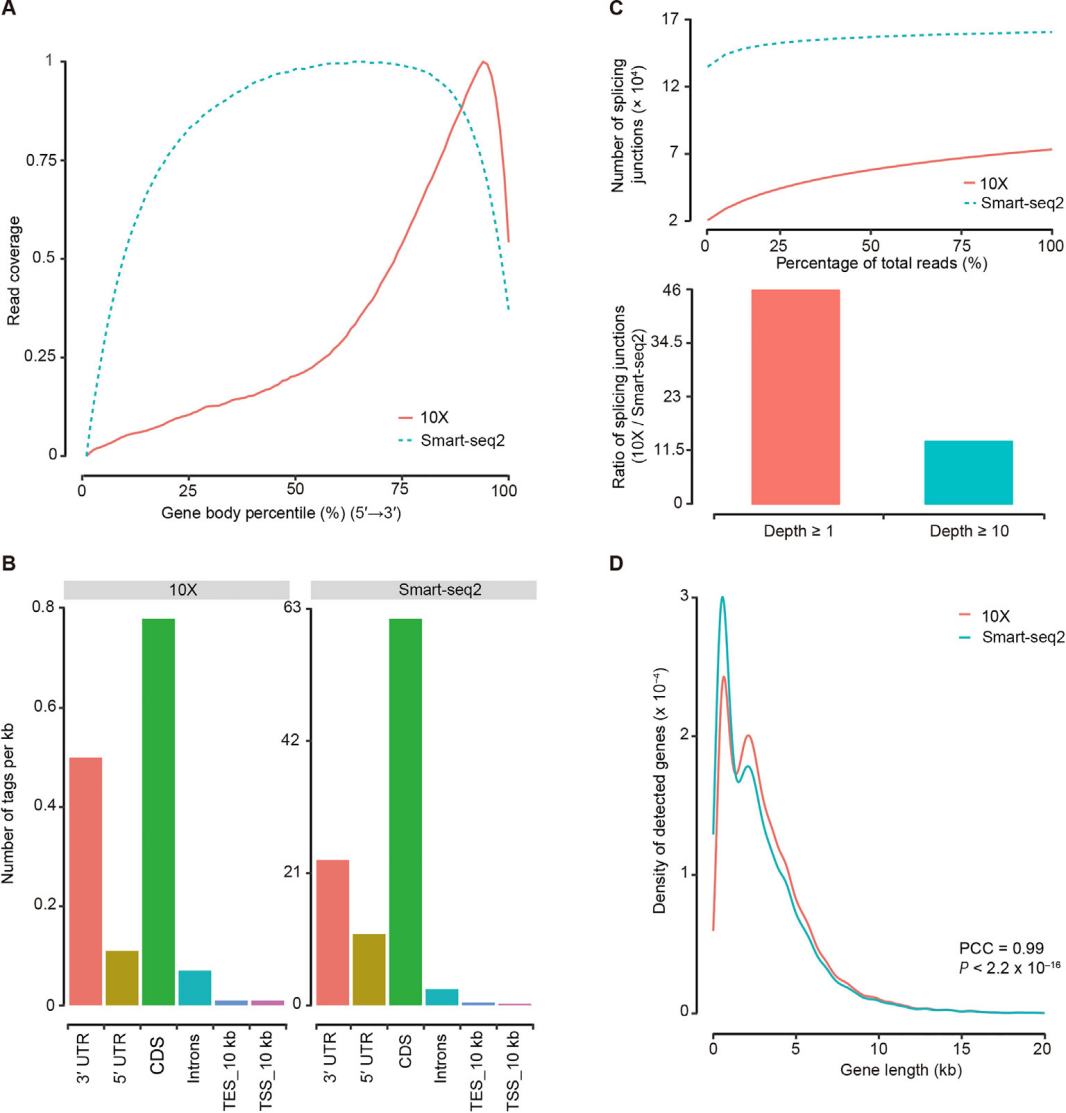
**Comparison of gene structural information** **A.** The read coverage over gene body detected in LT. **B.** Read distribution in genome detected in LT. **C.** Detection of known splice junctions in LT. **D.** Gene length was divided into consecutive 100 bins, we counted the number of detected genes in each bin, PCC values of gene number between Smart-seq2 and 10X were calculated in LT. UTR, untranslated region; CDS, coding sequence; TES_10 kb, 10 kb downstream of transcription end site; TSS_10 kb, 10 kb upstream of transcription start site; PCC, Pearson correlation coefficient.

**Supplementary Figure S8:**
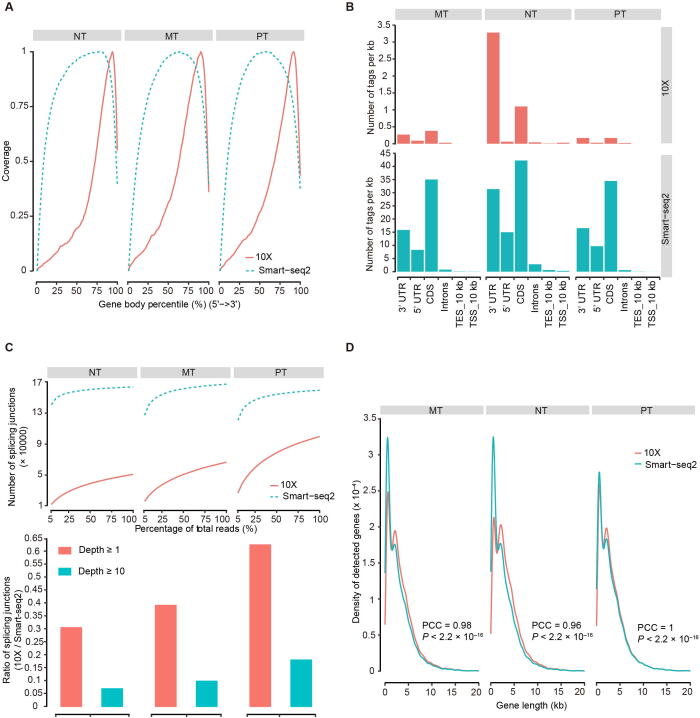
**Comparison of 3’-end VS full-length capture strategy A.** Reads coverage over gene body. **B.** Reads distribution in genome. **C.** Detection of known splice junctions. **D.** PCC of gene number in consecutive 100 bins divided by gene lengths between Smart-seq2 and 10X. PCC, Pearson correlation coefficient.

We finally evaluated how each of the two platforms captures the gene structural information. We first confirmed that the 10X reads showed a strong bias toward the 3′ ends of mRNAs as expected, while Smart-seq2 reads were more uniformly distributed in the gene bodies (Figure 6A and B, Figure S8A and B). For Smart-seq2, our sequencing depth was adequate for junction detection, evidenced by the number of detected known junctions reaching a plateau (Figure 6C, Figure S8C). The 10X data were not equipped for alternative splicing analysis due to the 3′-bias (Figure 6C, Figure S8C). Nevertheless, 10X still detected non-negligible number of junctions, even though they only accounted for approximately 50% of those junctions detected by Smart-seq2. Although Smart-seq2 data were clearly much more suitable for alternative splicing studies [41], [42], the limited number of splicing junctions detected by 10X might be suitable for certain analyses that rely on junction-based characterization, such as the RNA velocity analysis [43].

To evaluate whether gene length would introduce any bias in either of the platforms, we examined the correlation between the two platforms in terms of gene length and expression level. All calculated PCCs were at least 0.99 for all tested samples (Figure 6D, Figure S8D), demonstrating that mRNA molecular quantification was not influenced by either full-length or 3′ capture strategies.

## Discussion

Here we comprehensively evaluated two scRNA-seq platforms: Smart-seq2 was more sensitive for gene detection, and 10X had more noise and higher dropout ratio. 10X could detect rare cell populations due to high cell throughput. Both platforms had similar results in unique mapping ratio and assigning cells into different cell cycle phase. Smart-seq2 had better performance in detection of genes with low expression levels, of splicing junctions, and of cell–cell interactions. In terms of defining HVGs and detecting DEGs, each platform showed unique strength with limited overlap and they could provide complementary information. However, some limitations should be acknowledged. Firstly, the analysis of dropout rates was influenced by the large difference in sequencing depth of those two platforms. Considering an intrinsic property of the two methods, we did not perform down-sampling to equal sequencing coverage. Secondly, we only sequenced 94–189 cells per sample with the Smart-seq2 protocol, which may reduce the power to detect groups of cells. As has been previously shown, Smart-seq2 libraries should contain about 70 cells per cluster to achieve decent power [44]. Thirdly, UMI counts and read counts have different mean distributions, namely the negative binomial model is better fit for UMI number, and zero-inflated negative binomial model for read counts [45], which may impair the CV measure because CV is linked to the mean gene expression levels. Lastly, we only compared a set of data; however, it is very rare to identify both the Smart-seq2 and 10X data on exact the same samples. In fact, we had to generate such data on our own to achieve the direct comparison goal, as we were not able to find other suitable data. However, the main results were concordant with other reports, regarding detection sensitivity, dropout ratio, and cell types detected [17], [46], [47].

The advantage of scRNA-seq crucially depends on two parameters: cell number and sample complexity. These two parameters can be designed and chosen based on study objectives. The cell number is a key determinant for dissecting the sample composition. In this study, several hundreds of cells could capture abundant, but not rare, cell types using Smart-seq2. Thousands of cells or more could capture unique cell subtypes in both Smart-seq2 and 10X. Thus, the range of cell number in our study is relevant for other studies. In a heterogenous cell population or tissue, 1000–2000 cells could be adequate for clustering to distinguish various cell states [48].

However, the cost still seriously restricts studies that involve a large number of cells [8]. It seems a now standard practice to investigate tens of thousands of cells in a published paper. The cost is certainly an important factor for the optimal selection of the cell number. Smart-seq2 is an efficient method to uncover an in-depth characteristic of a rare cell population such as germ cells, without restriction from cell size, shape, homogeneity, and number. However, its overall cost is very high, and the laborious nature and technical variability can be intimidating because the reactions are carried out in individual wells for Smart-seq2 [44]. The huge advantage of 10X is the low cost and high throughput, making it better for complex experiments such as multiple treatments. Although many cells of each sample were added to each channel for 10X in our study, we just obtained 746, 1305, 1338, and 5282 cells by CellRanger (version 2.2, http://www.10xgenomics.com/). 10X cannot guarantee the yield of cells, and cell number may fluctuate wildly among experiments. For example, 60–4930 cells among 68 samples [49], and 1052–7247 cells among 25 samples [50] were obtained in two reports, respectively. The huge variability may come from tissue/cell types, inaccurate estimation of input cell number, or poor conditions and death of cells during experiments. A small number of cells cannot represent the biological image well [51]. Therefore, the trade-off between Smart-seq2 and 10X should be carefully assessed depending on data throughput and ultimate study objectives.

Samples generally contain a mixture of cells at various phases. However, effects of cell cycle may not be eliminated by directly discarding marker genes, as they can influence many other genes [52], [53]. To date, our results demonstrated that Smart-seq2 and 10X have similar power in assigning cells into different cyclic phases.

The scRNA-seq offers a much better biological resolution than bulk RNA-seq, with a cost of enhanced noise [54]. Reliable capture of mRNA molecules into cDNA is a challenge for lowly expressed genes in a single cell, which augments the probability of dropout events. This is more noticeable in 10X (Figure 5C). Moreover, 10X may acquire a few ambient transcripts that float in droplet because of cell lysis/death [19], which also results in noise; however, increased capture of single cells could compensate the inefficacy brought by noise and provide a more robust clustering. By contrast, Smart-seq2 had less noise and higher sensitivity but high cost, therefore the sample size attribute in Smart-seq2 and 10X should be established on rigorous design and well-defined rationale.

## Materials and methods

### Sample collection and single-cell processing

Tumor tissues of two donors were obtained from about 2 cm far from tumor edge, and adjacent normal liver tissues were located at least 2 cm far from the matched tumor tissue. Those fresh tissues were cut into pieces about 1 mm^3^ and digested with MACS tumor dissociation kit for 30 min. Suspended cells were filtered with 70-μm Cell-Strainer (Catalog No. 352350, BD, Franklin Lakes, NJ) in the RPMI-1640 medium (Catalog No. 0045092EF, Invitrogen, Carlsbad, CA) and then centrifuged at 400 *g* for 5 min, and the supernatant was discarded. To lyse red blood cells, pelleted cells were suspended in red blood cell lysis buffer (Catalog No. R1010, Solarbio, Beijing, China) and incubated on ice for 2 min. Finally, cell pellets were resuspended in sorting buffer after washed twice using 1× PBS.

### scRNA-seq

Based on FACS analysis (BD Aria III instrument), we used CD45 antibody (Catalog No. 11-0459, eBioscience, San Diego, CA) to separate CD45^+^ and CD45^−^ cells. Cells were sorted into 1.5-ml low binding tubes (Catalog No. 0030108051, Eppendorf, Saxony, Germany) with 50 ml sorting buffer, and into 96-well plates (Catalog No. PCR-96-FS-CS, Axygen, Union City, CA) with lysis buffer, which contained 1 μl 10 mM dNTP mix (Catalog No. 18427013, Fermentas, Glen Burnie, MD), 1 μl 10 μM Oligo(dT) primer, 1.9 μl 1% Triton X-100 (Catalog No. T8787, Sigma, St Louis, MO), and 0.1 μl 40 U/μl RNase inhibitor (Catalog No. 2313A, Takara, Dalian, China).

For 10X, single cells were processed with the GemCode Single Cell Platform using the GemCode Gel Bead, Chip and Library Kits (10X Genomics, Pleasanton, CA) following the manufacturer’s protocol. Samples were processed using kits pertaining to the V2 barcoding chemistry of 10X Genomics. Estimated 10,000 cells were loaded to each channel with the average recovery rate of 2000 cells. Libraries were sequenced on Hiseq 4000 (Illumina, San Diego, CA).

For Smart-seq2, transcript reverse transcription and amplification were performed following the protocol of Smart-seq2. We purified the amplified cDNA using 1× Agencourt XP DNA beads (Catalog No. A63881, Beckman, Pasadena, CA), and then performed quantification of cDNA of every cell with qPCR of *GAPDH* and fragment analysis with fragment analyzer AATI. To exclude short fragments (< 500 bp), cDNA products with high quality were further cleaned using 0.5× Agencourt XP DNA beads (A63881, Beckman). The concentration of each sample was quantified with the Qubit HsDNA Kit (Catalog No. 12640ES60, Invitrogen). Libraries were constructed with the TruePrep DNA Library Prep Kit V2 (Catalog No. TD501-01, Vazyme Biotech, Nanjing, China), and sequenced on Hiseq 4000 (Illumina, San Diego, CA) in paired-end 150 bp.

### Bulk RNA isolation and sequencing

After surgical resection, tissue was firstly stored in RNAlater RNA stabilization reagent (Catalog No. 76106, QIAGEN, Dusseldorf, Germany) and kept on ice. Total RNA was extracted with the RNeasy Mini Kit (Catalog No. 74104, QIAGEN) following the manufacturer’s instructions. Concentration of RNA was quantified with the NanoDrop instrument (ND-2000, ThermoFisher Scientific, Waltham, MA), and quality of RNA was evaluated with fragment analyzer (AATI, Palo Alto, CA). Libraries were constructed using NEBNext Poly(A) mRNA Magnetic Isolation Module Kit (Catalog No. E7490L, NEB, Ipswich, MA) and NEBNext Ultra RNA Library Prep Kit (Catalog No. E7770, NEB), and sequenced on Hiseq 4000 (Illumina) in paired-end 150 bp.

### Data reference

We used the GRCH38 human genome assembly as reference, which was downloaded from the Ensembl database (Ensembl 88; http://asia.ensembl.org). The PC genes and lncRNAs were categorized according to an Ensembl GTF file. Among those non-coding genes, rRNAs, tRNAs, miRNAs, snoRNAs, snRNA, and other known classes of small RNAs were discarded, and lncRNAs were defined as all non-coding RNAs longer than 200 nt and not classified to other RNA categories.

We retrieved the signature genes (extracellular region, cytoplasm, mitochondrion, ribosome, apoptotic process, metabolic process, membrane, and cell cycle) from the Gene Ontology (GO) database (GO:0005576, GO:0005737, GO:0005739, GO:0005840, GO:0006915, GO:0008152, GO:0016020, and GO:0007049, respectively; http://geneontology.org/). A list of human TFs was downloaded from the “Animal Transcription Factor Database” (http://bioinfo.life.hust.edu.cn/AnimalTFDB/).

### Quality control for scRNA

For Smart-seq2, sequenced reads were mapped to GRCH38 using the STAR aligner (version 2.6.0a) with the default parameters. These uniquely mapped reads in the genome were used, and multiplely mapped reads were discarded. Gene expression was quantified in counts using featureCounts (version 1.6.2; http://subread.sourceforge.net/), with parameters as follows: -T 2 -p -t exon -g gene_id. TPM values were derived from counts and calculated by: TPM = (10^6^ × C_ij_/L_i_)/(∑_i_C_ij_/L_i_), in which C_ij_ was count value of gene i in cell j and L_i_ was the length of gene i. Genes expressed (TPM > 0) in less than 10 cells were filtered out. Cells were removed according to the following criteria: 1) cells had fewer than 800 genes; 2) cells had over 50% reads mapped to mitochondrial genes.

For 10X, an expression matrice of each sample was obtained using the CellRanger toolkit (version 2.2; https://www.10xgenomics.com/) with the default parameters. Genes presented (UMI > 0) in less than 10 cells were filtered out. Cells were removed according to the following criteria: 1) cells had fewer than 500 genes; 2) cells had fewer than 900 UMI or over 8000 UMI; and 3) cells had more than 20% of mitochondrial UMI counts.

### CV

The CV is a standardized measurement of dispersion of a probability or frequency distribution. It is defined as the ratio of the standard deviation (SD) to the mean, namely CV = 100 × SD/mean.

### Cell cycle

We used the reported method [23] to categorize cells into cell cycle phases. Cells were classified in G1 phase if the G1 score is above 0.5 and larger than the G2/M score; in G2/M phase if the G2/M score is above 0.5 and larger than the G1 score; and in S phase if neither score is above 0.5 [55].

### Read distribution in genome and junction detection

To demonstrate the bias of read distribution in genome, we calculated read distribution over genome features, including coding sequence (CDS), 5′ untranslated region (5′ UTR), 3′ UTR, intron, 10 kb upstream of transcription start site (TSS_10 kb), and 10 kb downstream of transcription end site (TES_10 kb). When genome features overlapped, they were prioritized as follows: CDS > UTR > Intron > others.

We assessed sequencing depth for splicing junction detection by randomly resampling total alignments with an interval of 5%, and then detected known splice junctions from the reference gene model in GTF format.

### Down-sampling of reads

We used the seqtk software (version 1.3; https://github.com/lh3/seqtk) to randomly sample the FASTQ files for each library from Smart-seq2 with the “seqtk sample” command, using the random seed set to 100. And, we set this equal to the average number of reads per cell from 10X, as follows: 59 K, 34 K, 92 K, and 20 K for LT, MT, NT, and PT, respectively. Each library was randomly sampled five times. We used the first down-sampled datasets to evaluate dropout ratios.

### Saturation analysis

We resampled a series of alignment subsets (5%, 10%−100%) and then calculated RPKM value to assess sequencing saturation, which had been described [56]. “Percent Relative Error” was used to measure how the RPKM estimated from subset of reads (RPKM_est_) deviates from real expression level (RPKM_real_). The RPKM estimated from total reads was used as approximate RPKM_real_: percent relative error = 100 × (|RPKM_est_ – RPKM_real_|)/RPKM_real_.

### Cell clustering

After filtration, standard scRNA-seq analysis (differential expression, marker gene detection, and clustering) was performed using the Seurat package (version 2.3) [33], from a merged expression matrice of four samples. In brief, gene expression was log-normalized by the “NormalizeData” function with a scale factor of 10,000. HVGs were calculated with the “FindVariableGenes” function with parameters “mean.function” = “ExpMean” and “dispersion.function” = “LogVMR”. The top 1000 genes in the “hvg.info” slot, which was decreasingly ordered based on dispersion, were selected as HVGs used in downstream analysis. Data were scaled with the “ScaleData” function using the selected HVGs, with the parameter “vars.to.regress” = c(“percent.mito”, “nUMI”) for 10X, and “vars.to.regress” = “percent.mito” for Smart-seq2. CCA was calculated using the “RunCCA” function, with the parameters “genes.use” = HVGs, “num.cc” = 30, which was used to remove batch effects of patients. The “AlignSubspace” function was then used to align subspaces across patients, with the parameter “dims.align” = 1:20, which was chosen by visualization plot of the “MetageneBicorPlot” function. Cells were clustered by the “FindClusters” function using the first 20 canonical correlations (CCs), with the resolution parameter set to 0.8 for 10X datasets and 1.2 for Smart-seq2 datasets. “RunUMAP” function was used with the parameters “reduction.use” = “cca.aligned” and “dims.use” = 1:20. DEGs and marker genes were detected using the “FindAllMarkers” function, with the parameters “logfc.threshold” = 0.25, “min.pct” = 0.25, and “test.use” = “MAST”. The *P* value was adjusted using Bonferroni correction, and DEGs were identified with the threshold of adjusted *P* < 0.01. Subsequently, cell clusters were annotated manually, according to known markers. Hepatocyte marker genes were *ALB* and *SERPINA1*; malignant cell marker genes were *STMN1*, *H2AFZ*, *CKS1B*, and *TUBA1B*; fibroblast marker genes were *RGS5* and *NDUFA4L2*; CAF marker genes were *LUM*, *SFRP4*, *DCN*, *FBLN1*, and *COL1A1*; myofibroblast marker genes were *MYH11*, *TAGLN*, and *ACTA2*; epithelial cell marker genes were *KRT18*, *KRT8*, and *EPCAM*; and endothelial cell marker genes were *ENG*, *PECAM1*, and *VWF*.

### CCA on an individual tumor from both technologies

For an individual sample, filtration and standard scRNA-seq analysis were performed as above mentioned. Except that the top 2000 genes in the “hvg.info” slot were selected as HVGs. Following the “MergeSeurat” function used to integrate samples from two technologies, the “ScaleData” and “RunCCA” functions were performed using shared genes of the top 2000 HVGs from each sample. Cells were clustered by the “FindClusters” function using the first 20 CCs, with the resolution parameter set to 1, 0.8, 0.5, and 0.5 for LT, MT, NT, and PT, respectively.

### Cell–cell interaction prediction

Cell–cell interaction prediction was performed by CellphoneDB (version 2.0) [37] using the log-normalized expression data. We performed pairwise comparisons between all three platform-shared cell types. The number of significant interacting gene pairs was identified with *P* < 0.01 as a cutoff.

### Data visualization and statistics

Microsoft R Open (version 3.5.1; https://mran.microsoft.com/) was used, and ggplot2 package (version 3.1.0) were used to generate data graphs. Data were presented as mean ± SD in figures. KEGG pathway enrichment (*P* < 0.01) were performed using clusterProfiler package (version 3.9.2) [57]. DEGs were identified with the “FindMarkers” function (“logfc.threshold” = 0.25 and “min.pct” = 0.25) using the MAST method [58], and *P* value was adjusted using Bonferroni correction, with the threshold of adjusted *P* < 0.01.

## Ethical statement

This study was approved by the Ethics Committee of Beijing Shijitan Hospital, Capital Medical University, China. All patients provided written informed consent for sample collection and data analysis.

## Data availability

The raw sequence data reported in this study have been deposited in the Genome Sequence Archive [59], at the National Genomics Data Center, Beijing Institute of Genomics, Chinese Academy of Sciences / China National Center for Bioinformation (GSA: HRA000063 and HRA000064), and are publicly accessible at http://bigd.big.ac.cn/gsa.

## Code availability

Code for clustering and plots is available on GitHub (https://github.com/Japrin). Other custom scripts are available upon request.

## CRediT author statement

**Xiliang Wang:** Conceptualization, Formal analysis, Writing - original draft, Data curation, Visualization. **Yao He:** Formal analysis, Data curation, Visualization. **Qiming Zhang:** Writing - original draft, Investigation, Resources. **Xianwen Ren:** Writing - review & editing. **Zemin Zhang:** Supervision, Writing - review & editing. All authors read and approved the final manuscript.

## Competing interests

The authors have declared no competing interests.

## Supplementary material

Supplementary Table S1 **Cell number of each sample**

Supplementary Table S2 **The average number of detected genes in every cell from Smart-seq2 after random down-sampling**

Supplementary Table S3 **List of the most highly expressed genes (Top10)**

Supplementary Table S4 **KEGG enrichment results of 10X-specific, bulk-specific, and Smart-seq2-specific genes in the top25% list**

Supplementary Table S5 **DEGs among samples with conflicting change trends**

Supplementary Table S6 **DEGs among cell types with conflicting change trends**

Supplementary Table S7 **The number of DEGs among cell types after CCA analyses on an individual sample**

Supplementary Table S8 **List of genes with zero dropout ratio in a sample**
