# Supplementary material for: Direct Comparative Analyses of 10X Genomics Chromium and Smart-seq2
Source: Genomics Proteomics Bioinformatics. 2021 Mar 2;19(2):253–66. doi: 10.1016/j.gpb.2020.02.005 (PMC8602399; doi:10.1016/j.gpb.2020.02.005)
Supplement: Supplementary Table S1 — Cell number of each sample [file mmc1.docx]

**Table S1 Cell number of each sample**

| **Sample** | **Tissue source** | **Diagnosis** | **10X** | **Smart-seq2** |
| --- | --- | --- | --- | --- |
| LT | Liver | Hepatocellular carcinoma | 1338 | 94 |
| MT | Liver | Rectal cancer with liver metastasis | 1305 | 183 |
| NT | Liver | Hepatocellular carcinoma | 746 | 189 |
| PT | Rectum | Rectal cancer with liver metastasis | 5282 | 135 |

*Note*: LT, liver tumor; MT, metastasized tumor; NT, adjacent non-tumor tissue; PT, primary tumor.
