# Supplementary material for: Direct Comparative Analyses of 10X Genomics Chromium and Smart-seq2
Source: Genomics Proteomics Bioinformatics. 2021 Mar 2;19(2):253–66. doi: 10.1016/j.gpb.2020.02.005 (PMC8602399; doi:10.1016/j.gpb.2020.02.005)
Supplement: Supplementary Table S2 — The average number of detected genes in every cell from Smart-seq2 after random down-sampling [file mmc2.docx]

**Table S2 The average number of detected genes per cell from Smart-seq2 after random down-sampling**

| **Sample** |  | **10X** |  | **Smart-seq2** | | | | |
| --- | --- | --- | --- | --- | --- | --- | --- | --- |
| **LT** |  | 2682 |  | 3078 | 3081 | 3082 | 3081 | 3084 |
| **MT** |  | 1853 |  | 2538 | 2542 | 2539 | 2541 | 2543 |
| **NT** |  | 2123 |  | 2279 | 2280 | 2278 | 2280 | 2272 |
| **PT** |  | 1104 |  | 1830 | 1832 | 1832 | 1831 | 1830 |

*Note*: Each library was randomly sampled five times.
