# Supplementary material for: Direct Comparative Analyses of 10X Genomics Chromium and Smart-seq2
Source: Genomics Proteomics Bioinformatics. 2021 Mar 2;19(2):253–66. doi: 10.1016/j.gpb.2020.02.005 (PMC8602399; doi:10.1016/j.gpb.2020.02.005)
Supplement: Supplementary Table S3 — List of the most highly expressed genes (Top10) [file mmc3.docx]

**Table S3 List of the most highly expressed genes (Top10)**

|  | **LT** | **MT** | **NT** | **PT** |
| --- | --- | --- | --- | --- |
| 10X | *MALAT1* | *MALAT1* | *IGKC* | *MALAT1* |
|  | *B2M* | *MT-CO1* | *IGLC2* | *IGFBP7* |
|  | *TMSB4X* | *PPY* | *IGHG4* | *MT-CO1* |
|  | *IGFBP7* | *MT-CO2* | *IGHG3* | *B2M* |
|  | *CPA1* | *MT-ND2* | *MALAT1* | *JUN* |
|  | *MT-RNR2* | *TTR* | *IGLC3* | *HSP90AA1* |
|  | *MT-CO3* | *MT-CO3* | *IGHA1* | *MT-ND4* |
|  | *MT-CO1* | *MT-ND4* | *IGHG1* | *RPL3* |
|  | *EEF1A1* | *CHGB* | *JCHAIN* | *MT-ND2* |
|  | *RPL41* | *MT-CYB* | *IGHM* | *EEF1A1* |
| Smart-seq2 | *MT-RNR2* | *MT-CO1* | *MT-RNR2* | *MT-RNR2* |
|  | *MT-CO1* | *MT-CYB* | *MT-CO1* | *MT-CYB* |
|  | *MT-CO3* | *MT-CO3* | *IGKC* | *MT-RNR1* |
|  | *MT-CYB* | *MT-RNR2* | *MT-CO3* | *MT-CO1* |
|  | *MT-RNR1* | *MT-CO2* | *MT-CYB* | *MT-CO3* |
|  | *MT-ATP6* | *MT-ND4* | *MT-ATP6* | *MT-ATP6* |
|  | *MT-ND2* | *MT-ND2* | *MT-ND4* | *MT-ND4L* |
|  | *CPA1* | *MT-ND5* | *MT-RNR1* | *MT-ND4* |
|  | *MT-ND4L* | *MT-ND4L* | *MT-ND2* | *MT-CO2* |
|  | *MT-ND4* | *MT-ATP6* | *MT-ND4L* | *MALAT1* |
| Bulk | *CPA1* | *MT-CO1* | *ALB* | *MT-CO1* |
|  | *MT-CO1* | *MT-ATP6* | *MT-CO1* | *MT-ND4* |
|  | *MT-ATP6* | *MT-RNR2* | *MT-ATP6* | *MT-CO2* |
|  | *MT-CO3* | *MT-CO3* | *MT-CO3* | *MT-ND2* |
|  | *MT-CO2* | *MT-ND4* | *MT-ND4* | *MT-ATP6* |
|  | *MT-ND4* | *MT-CO2* | *MT-CO2* | *MT-CO3* |
|  | *MT-RNR2* | *MT-CYB* | *MT-RNR2* | *MT-ND3* |
|  | *CLPS* | *MT-ND1* | *MT-ND6* | *MT-CYB* |
|  | *MT-CYB* | *MT-ND4L* | *MT-CYB* | *MT-RNR2* |
|  | *MT-ND1* | *MT-ND2* | *MT-ND1* | *PCSK1N* |
