# Supplementary material for: Direct Comparative Analyses of 10X Genomics Chromium and Smart-seq2
Source: Genomics Proteomics Bioinformatics. 2021 Mar 2;19(2):253–66. doi: 10.1016/j.gpb.2020.02.005 (PMC8602399; doi:10.1016/j.gpb.2020.02.005)
Supplement: Supplementary Table S5 — DEGs among samples with conflicting change trends [file mmc5.docx]

**Table S5 DEGs among samples with the change trends conflicting**

| **Sample** | **Change type** | **Genes** |
| --- | --- | --- |
| LT | 10X_Up & Smart-seq2_Down | *GNG11* |
|  | 10X_Down & Smart-seq2_Up | *LPP*, *ZFP36L1*, *TPM2*, *UBB*, *HEYL*, *CPM*, *ADAMTS9*, *SPTSSA*, *RPSA*, *RGS16*, *TAGLN* |
| MT | 10X_Up & Smart-seq2_Down | *RPL31*, *RPL34*, *RPS15*, *RPS28*, *PDCD5*, *NOP10*, *RPS13*, *USMG5*, *UQCRH*, *TMA7*, *PFDN5*, *RPL27*, *RPS12*, *RPL23*, *RPS19*, *ATP5J*, *NDUFB3*, *RPL12*, *RPL18*, *RPL9*, *HIST1H4C*, *RPL27A*, *RPS14*, *RPS27*, *RPS11*, *RPL35*, *RPL39*, *RPS20*, *RPLP1*, *S100A6*, *NDUFS5*, *COX6A1*, *NDUFA1*, *OST4*, *RPLP2*, *COX8A*, *COX7C*, *COX6B1*, *NDUFB7*, *RPS18*, *NDUFS6*, *RPS8*, *COX7A2*, *COX5B* |
|  | 10X_Down & Smart-seq2_Up | *NEFM*, *EIF4A3* |
| NT | 10X_Up & Smart-seq2_Down | *RPL9P9* |
|  | 10X_Down & Smart-seq2_Up | *TSC22D3*, *KLF9*, *PDK4* |
| PT | 10X_Up & Smart-seq2_Down |  |
|  | 10X_Down & Smart-seq2_Up | *RPL9P9* |
