# Supplementary material for: Direct Comparative Analyses of 10X Genomics Chromium and Smart-seq2
Source: Genomics Proteomics Bioinformatics. 2021 Mar 2;19(2):253–66. doi: 10.1016/j.gpb.2020.02.005 (PMC8602399; doi:10.1016/j.gpb.2020.02.005)
Supplement: Supplementary Table S6 — DEGs among cell types with conflicting change trends [file mmc6.docx]

**Table S6 DEGs among cell types with conflicting change trends**

| **Cell type** | **Change type** | **Genes** |
| --- | --- | --- |
| Endothelial cell | 10X_Up & Smart-seq2_Down | *DNASE1L3* |
|  | 10X_Down & Smart-seq2_Up | *ADAMTS1* |
| Epithelial cell | 10X_Up & Smart-seq2_Down | *RPL31*, *RPS15*, *RPS13*, *NDUFS5*, RPL34, *RPLP2*, *RPL35*, *RPS20*, *RPL27A* |
|  | 10X_Down & Smart-seq2_Up |  |
| Fibroblast | 10X_Up & Smart-seq2_Down |  |
|  | 10X_Down & Smart-seq2_Up | *AKAP12*, *MT-RNR2*, *RPL35* |
